# Frequency of hypovitaminosis D and its associated risk factors in newly diagnosed pulmonary tuberculosis patients

**DOI:** 10.12669/pjms.322.8996

**Published:** 2016

**Authors:** Fahad Azam, Abida Shaheen, Rabia Arshad

**Affiliations:** 1 Dr. Fahad Azam, Shifa College of Medicine, Shifa Tamer e Millat University, Islamabad, Pakistan; 2 Dr. Abida Shaheen, Shifa College of Medicine, Shifa Tamer e Millat University, Islamabad, Pakistan; 3 Dr. Rabia Arshad, Altamash Institute of Dental Medicine, Karachi, Pakistan

**Keywords:** Tuberculosis, Vitamin D, Insufficiency, Deficiency, Associated risk factors

## Abstract

**Objective:**

To find out the frequency of hypovitaminosis D and its associated risk factors in newly diagnosed pulmonary tuberculosis patients prior to administration of standard anti tuberculosis therapy.

**Methods:**

This cross-sectional study was carried out in Ojha Institute of Chest Diseases-DUHS. After approval from BASR and following written informed consent eighty newly diagnosed, as per WHO criteria, tuberculosis patients were enrolled. Prior to the initiation of anti tuberculosis therapy, the serum vitamin D level was determined by 25-OH Vitamin D kit using the chemiluminescent immunoassay (CLIA) method. A cut off value of >30 ng/mL of serum vitamin D was taken as normal whereas a range between 10-30 ng/mL and <10 ng/mL were considered insufficient and deficient respectively. Frequency of socio-demographic associated risk factors of hypovitaminosis D was also determined.

**Results:**

Out of eighty newly diagnosed tuberculosis patients 33 (41.25%) were males and 47(58.75%) were females with their ages ranging from 18-50 years. 54 patients (26 male and 28 female patients) were smokers. BMI of all the patients was found to be less than the normal ranges. Hypovitaminosis was present in all the cases. Vitamin D insufficiency was found in 49 participants (20 male and 29 female) whereas 31 patients (13 male and 18 female) were found to be vitamin D deficient.

**Conclusion:**

Prevalence of serum vitamin D level derangement is very high in newly diagnosed patients with pulmonary tuberculosis in our local setting which necessitates administration of adjuvant vitamin D along with standard anti tuberculosis therapy.

## INTRODUCTION

Tuberculosis, a disease spread by obligate intracellular organism *Mycobacterium tuberculosis*, is probably one of the oldest diseases known to mankind. Tuberculosis is an important issue of concern for public health globally and in the year 2013, nine million new cases of tuberculosis and 1.5 million deaths were reported worldwide due to it.^1^ In Pakistan, tuberculosis has been an important area for concern because of its increasing prevalence. According to the WHO, in 2013, the prevalence of all forms of tuberculosis in Pakistan was reported to be 275 cases per 100,000 population and currently Pakistan ranks fifth among countries worst-affected by the disease.^2^ Even though the disease is curable, the treatment is long and has risks of treatment failure or relapse. This highlights the need for prevention of tuberculosis by avoiding or correcting the causative and predisposing factors for tuberculosis such as poor nutrition, lack of sun exposure, unhygienic living conditions and low immunity.^3^

In recent years there has been escalating evidence which highlights the significance of vitamin D role in the response of immune system against mycobacterium tuberculosis which is an important predictor in the possible outcomes of tuberculosis.^4^ There is up regulation of expression of vitamin D receptor and 25-hydroxyvitamin D-1α-hydroxylase after the activation of a macrophage or monocyte through stimulation of its toll-like receptor 2/1 (TLR2/1) by *Mycobacteriumtuberculosis* or otherinfectious agents. Research suggests that levels of 25-hydroxyvitamin D [] upto 30 ng/ml or higher provides adequate substrate for conversion of 25(OH) D by 1-OHase to its active form, 1,25 dihydroxyvitamin D [1,25(OH)_2_D], which thereafter is able to penetrate into the nucleus, to eventually enhance the expression of cathelicidin which is an anti mycobacterial peptide.^5^, ^6^

If levels of 25(OH) D in serum are below 20 ng/ml, ability of the monocyte or macrophage to initiate this innate immune response is greatly compromised. Furthermore, the enhanced synthesis of 1,25(OH)_2_D in monocytes and macrophages results in increased ability of activated T lymphocytes to act locally and enhance synthesis of cytokine as well as activate B lymphocytes, which regulate immunoglobulin synthesis.^7^, ^8^ Therefore the factors which contribute towards low plasma levels of Vitamin are also documented to promote and predispose to pulmonary TB.

The role of host immunity and its influence on the host-pathogen interaction is very important in the possible outcomes after being infected by mycobacterium tuberculosis. The relationship between hypovitaminosis D and predisposition to tuberculosis has been observed in England and African countries^9^, ^10^ but documented studies for Pakistan are scarce. The aim of present study was to find out the frequency of hypovitaminosis D and its associated risk factors in newly diagnosed pulmonary tuberculosis patients prior to administration of standard anti tuberculosis therapy.

## METHODS

### Subjects

Eighty newly diagnosed patients having pulmonary tuberculosis were enrolled from the OPD of Ojha Institute of Chest Diseases, Dow University of Health Sciences (DUHS) from November 2010 to March 2011. The patients were of either sex with ages ranging between 18-50 years. They were diagnosed according to the WHO criteria for pulmonary tuberculosis which is Two sputum smear examinations on direct smear microscopy positive for acid fast bacilli (AFB+); one sputum examination positive for AFB and radiographic abnormalities consistent with active pulmonary tuberculosis; or one sputum specimen positive for AFB and one culture positive for AFB. Patients with AFB sputum smear negative; other concomitant ailments such as chronic kidney disease, liver problems, pulmonary silicosis, patients already on anti tuberculosis medication, taking Vitamin D, pregnant or lactating women were excluded from the study.

### Study design

It was a cross-sectional hospital based study which was conducted after obtaining approval from IRB (DUHS) (Reference number: IRB-165/DUHS-10) and BASR of DUHS (reference number: DUHS/DR/2010/485). After a written informed consent all newly diagnosed pulmonary tuberculosis patients were subjected for evaluation of serum vitamin D levels. Prior to commencing anti tuberculosis therapy, from each patient 10 ml blood sample was collected after an overnight fast and serum Vitamin D level was evaluated by using electrochemiluminescence immunoassay on a Roche Elecsys 10100/201 system. Sociodemographic features were recorded in a specially designed proforma.

### Statistical analysis

Data was gathered through a detailed proforma completed for each case and ratios, percentages and mean ± SD were calculated through SPSS program 17. P value less than 0.05 was taken statistically significant.

## RESULTS

Out of 80 study participants, 33 were males and 47 were females. Age ranged from 18 to 50 years with mean age 33.33±12.21 years. Average age of males was 35.03±10.11 and average age of females was 31.93±11.37 years. The average BMI in male patients was 18.1±0.3 and the average BMI in females was found to be 17.53±0.27. Occupation wise five were drivers, six were laborers, three were cooks, twelve were domestic servants, thirty two were housewives, two were government servants, eight were teachers and twelve were jobless. Moreover, 80% of male patients and 16% of female patients were smokers prior to diagnosis of tuberculosis.

Hypovitaminosis was present in all the cases. The prevalence of Vitamin D insufficiency and deficiency was found to be 60.60% and 39.39% in male tuberculosis patients and 61.70% and 38.29% in female tuberculosis patients respectively. The vitamin D levels in male and female study participants with respect to insufficiency and deficiency are provided in Table-I. The socio-demographic features of study participants are given in Table-II. Levels of serum 25(OH)D observed in different socio-demographic variables are presented in Table-III.

**Table-I:** Hypovitaminosis D in male and female study participants

Vitamin D levels	Gender		*p-value*

	Men (percentage)	Women (percentage)	
Insufficiency (49 patients) (61%)	20 (40.81)	29 (59.18)	(0.039)
Deficiency (31 patients) (39 %)	13 (41.93)	18 (58.06)	(0.042)

**Table-II:** Socio-demographic associated risk factors

Variables	Frequency	Percentage (%)
*Gender*		
Male	33	41.25
Female	47	58.75
Age		
*<20*	39	48.75
20-30	20	25
30-40	13	16.25
40-50	8	10
*BMI*		
<16	45	56.25
16-17	16	20
17.18.5	18	22.5
18.5-25	1	1.25
*Level of Education*		
Illiterate	55	68.75
Literate	25	31.25
a)Primary level	a) 18	a)72
b)Secondary level	b) 07	b) 28
*Occupation*		
House wife	32	40
Laborer	6	7.5
Driver	5	6.25
Cook	3	3.75
Domestic servant	12	15
Teacher	8	10
Government Servant	2	2.5
Jobless	12	15
*Ethnicity*		
Punjabi	7	8.75
Pushto	10	12.5
Hazarwal	09	11.25
Siraiki	24	30
Sindhi	14	17.5
Kashmiri	07	8.75
Urdu speaking	07	8.75
Bengali	02	2.5
*SES*		
less than 6,000 per month	61	76.25
6,000 to 25,000 per month	19	23.75
*Smoking status*		
Yes	42	52.5
No	38	47.5

**Table-III:** Serum 25(OH)D by category of sociodemographic variables among TB patients.

Variable	n (%)	Serum 25(OH)D, ng/ml	*p- value*
*Gender*			
Male	37 (46.25) *	19.45 *	0.002
Female	43 (53.75)	9.41	
*Age*			
<20	39 (48.75)	8.34	0.2
20-30	20 ((25)	12.22	
30-40	13 (16.25)	13.34	
40-50	8 (10)	9.13	
*Ethnicity*			
Punjabi	07 (8.75)	8.22	
Pushto	10 (12.5)	11.34	
Hazarawal	09 (11.25)	9.45	
Siraiki	24 (30) *	9.33 *	0.045
Sindhi	14 (17.5)	12.38	
Kashmiri	07 (8.75)	12.5	
Urduspeaking	07 (8.75)	10.76	
Bengali	02 (2.5)	10.88	
*Occupation*			
House wife	32 (40) *	9.87 *	
Laborer	6 (7.5)	12.34	
Driver	5 (6.25)	10.11	
Cook	3 (3.75)	11.1	0.001
Domestic servant	12 (15)	12.29	
Teacher	8 (10)	10.55	
Government Servant	2 (2.5)	9.76	
Jobless	12 (15	8.76	
*Marital status*			
Married	44 (55)	12.33	0.21
Single	36 (35)	11.34	
Separate/Widowed	0		
*SES*			
less than 6,000 per month	61 (76)	12.37	0.018
6,000 to 25,000 per month	19 (24)	15.11	
*Smoking*			
Yes	42 (52.5)	13.97	0.85
No	38 (47.5)	14.35	
*BMI kg/m2*			
<16	45 (56.25) *	9.89 *	0.028
16-17	16 (20)	12.7	
17.18.5	18 (22.5)	11.41	
18.5-25	1 (1.25)	11.05	

* = statistically significant

One way ANOVA test

## DISCUSSION

In this study, we observed Vitamin D deficiency in newly diagnosed pulmonary tuberculosis patients. The high prevalence of vitamin deficiency seen among our study population could be due to nutritional factors as majority of the patients belonged to poor socioeconomic status. However, it is highly unlikely that poor nutrition could be the only reason for this deficiency as it is a well-known fact that only ten percent of the Vitamin D is obtained from diet and the rest (90%) of it is produced in the skin under the influence of ultraviolet sunlight of the sun. Thus, only nutritional aspects are not likely the basis of the high frequency of low vitamin D in TB patients.^11^, ^12^

The first reports about the possibility of relationship between vitamin D and tuberculosis surfaced twenty years ago,^13^ but since then there have been conflicting reports about any such association in the subsequent studies. A number of studies conducted on Asian and African immigrants in England, African immigrants living in Australia, Ugandian and Korean population have reported very low levels of 25(OH)D and higher prevalence of vitamin D deficiency in TB patients than non-TB individuals.^9^, ^10^, ^14^, ^15^ However, there was contrasting evidence in studies conducted in Tanzania and Vietnam, which showed no considerable difference in 25(OH)D levels between TB cases and matched controls.^16^, ^17^

Vitamin D deficiency could be an antecedent risk factors for TB in a similar manner as the disease itself could lead to low 25(OH)D levels in TB patients. The correlation between vitamin D and TB is mediated through increased production of cathelicidin and localized action of the 1,25(OH)_2_D producedin monocytes or macrophages on activated B lymphocytes regulating immunoglobulin production activated T lymphocytes controlling cytokine synthesis.^6^

In our study, mean BMI for both male and female tuberculosis patients was below normal levels that is an indicator of the meager health and nutritional condition of these participants. None of the patients in the study had white collar jobs and all the patients belonged to lower SES. Sixty four (80%) tuberculosis patients were extremely poor and earned less than 6,000 rupees per month which further confirms the poor nutritional status and poor hygienic conditions of these patients. The positive connection between BMI and serum 25(OH)D in our study isnot similar to negative relationships observed in studies conducted in western countries which are elucidated by sequestration of fat soluble vitamin D in adipose tissue. We suggest that the positive association between the two variables is due to the reason that low BMI in these patients reveals a prolonged period of less sun exposure and hence, decreased vitamin D status.^18^

In male tuberculosis patients vitamin D insufficiency (52.63%) was slightly more common than Vitamin D deficiency (47.36%). In contrast, in female patients, vitamin D deficiency was 2.2 times more prevalent than vitamin D insufficiency. This could be a result of the poor sun exposure in female tuberculosis patients because of social and cultural reasons^11^ as most of them were house wives and wore veil (burqa) whereas most of the male patients had moderate sun exposure.

In a study conducted in Egypt, plasma levels of Vitamin D were found to be low at the time of diagnosis of pulmonary TB which decreased further at four and six months during treatment. 19 These findings are in accordance with our results. Furthermore, in similar studies conducted in northern India^20^ and rural areas around Lahore, Pakistan^21^, prevalence of Vitamin D deficiency was higher; and Vitamin D insufficiency was less compared to our findings. A reason for this finding could be due to the fact that both quoted studies study were conducted in very poor rural population where poor nutrition and very low BMI was reported. In another study reported in Tanzania, Vitamin D levels in TB patients were again found to be considerably lower than matched controls but plasma levels of Vitamin D were found to be negatively associated with weight and BMI of the TB patients.^16^ This finding was in contrast to our findings where plasma levels of Vitamin D were positively associated with the weight and BMI of the TB patients. Reason for this difference could be the fact that low BMI in our population was due to malnutrition whereas weight loss in the study conducted in Tanzania was artificially induced through surgical means which could have resulted in less sequestration of the fat soluble vitamin after the removal of excessive fat.

In our study, all patients showed deranged Vitamin D levels prior to the administration of standard anti tuberculosis therapy which means that this derangement was not drug induced.

The fact that matched controls were not enrolled for comparison of the relevant variables can be considered a limitation of the study. An association of low plasma levels of Vitamin D with pulmonary tuberculosis was found but a causal relationship could not be established which is another limitation of our study.

## CONCLUSION

Frequency of hypovitaminosis D is found to be 100% with insufficiency (61.25%) and deficiency (38.75%) in newly diagnosed pulmonary tuberculosis patients. Whereas gender, ethnicity, occupation and BMI were the risk factors associated with it. The fact that all tuberculosis patients showed derangements in vitamin D levels implies that vitamin D should be administered as adjuvant therapy with standard anti tuberculosis therapy. This might be helpful in reducing the severity of disease and preventing complications.
